# Changes in COVID-19 outbreak severity and duration in long-term care facilities following vaccine introduction, England, November 2020 to June 2021

**DOI:** 10.2807/1560-7917.ES.2021.26.46.2100995

**Published:** 2021-11-18

**Authors:** Rebecca Giddings, Maria Krutikov, Tom Palmer, Christopher Fuller, Borscha Azmi, Madhumita Shrotri, Aidan Irwin-Singer, Gokhan Tut, Paul Moss, Andrew Copas, Laura Shallcross

**Affiliations:** 1 UCL Institute of Health Informatics, London, United Kingdom; 2 UCL Institute for Global Health, London, United Kingdom; 3 Department of Health and Social Care, United Kingdom; 4 Institute of Immunology and Immunotherapy, University of Birmingham, Birmingham, United Kingdom

**Keywords:** COVID-19, vaccination, immunization, outbreak, care home

## Abstract

We describe the impact of changing epidemiology and vaccine introduction on characteristics of COVID-19 outbreaks in 330 long-term care facilities (LTCF) in England between November 2020 and June 2021. As vaccine coverage in LTCF increased and national incidence declined, the total number of outbreaks and outbreak severity decreased across the LTCF. The number of infected cases per outbreak decreased by 80.6%, while the proportion of outbreaks affecting staff only increased. Our study supports findings of vaccine effectiveness in LTCF.

Long-term care facilities (LTCF) have been disproportionately affected by the coronavirus disease (COVID-19) pandemic, with residents particularly at risk of severe outcomes [1]. English LTCF have been impacted by three waves of severe acute respiratory syndrome coronavirus 2 (SARS-CoV-2) infections, with changes in disease control measures and epidemiology of infection over successive waves. The aim of this study was to describe how the changing epidemiology and vaccination introduction have affected COVID-19 outbreak characteristics, including outbreak duration and severity, in LTCF to inform revaccination strategies and the implementation of non-pharmaceutical interventions to prevent transmission.

## Identifying outbreaks in long-term care facilities

The VIVALDI study, established in May 2020, is a prospective cohort study of LTCF staff and residents from a range of providers in England which provide care to adults aged over 65 years, with participant follow-up for up to 18 months [2]. The PCR and lateral flow device (LFD) results from March 2020 onwards were linked at individual and LTCF level (see Supplement for data linkage methodology).

Within this cohort, we explored the characteristics of COVID-19 outbreaks during different time periods to account for vaccination roll-out and COVID-19 pandemic waves in England: (i) 1 November 2020 to 31 December 2020 (second wave, pre-vaccination), (ii) 1 January 2021 to 28 February 2021 (second wave, low first dose vaccination coverage), (iii) 1 March 2021 to 30 April 2021 (second wave, high first dose vaccination coverage, low second dose vaccination coverage) and (iv) 1 May 2021 to 30 June 2021 (third wave, high first and second dose vaccination coverage).

We extracted LTCF characteristics from seven datasets (see Supplementary Table S1) and assessed them mid-way through each time period.

Long-term care facilities commenced regular SARS-CoV-2 PCR testing of staff (weekly) and residents (monthly) in the whole facility in July 2020 in addition to whole-LTCF (staff and residents) testing during a COVID-19 outbreak [3]. In December 2020, additional LFD testing was introduced for staff (twice weekly) and increased staff and resident testing during an outbreak [4]. For this analysis, outbreaks were defined as two or more positive SARS-CoV-2 tests (PCR/LFD) in the same LTCF within a 14-day period and ended when no further positive tests were reported for 28 days following the last positive [5]. Outbreak severity markers were outbreak length (days), number of people infected and number of deaths confirmed as related to COVID-19 during the outbreak period (Supplementary Table S1). A large outbreak was defined as infection of at least one third of the LTCF staff and residents.

## Characteristics of long-term care facilities

A total of 330 LTCF were included in the study, representing a mix of for-profit and not-for-profit LTCF across England (Table 1). The median number of staff per LTCF was 45 (interquartile range (IQR): 33–61, range: 1–156) across the time periods and the median number of residents was 36 (IQR: 26–48, range: 1–149). The majority of LTCF were medium-sized with 50–100 individuals (staff and residents) (range: 8–304) and had a care quality commission (CQC) rating of ‘good’. Median first dose vaccination coverage per LTCF was consistently higher in residents than staff: 85.7% (IQR: 67.7–93.1) vs 57.4% (IQR: 39.9–75.6) between January and February 2021, with coverage rising to 90.3% in residents (IQR: 79.3–95.7) and 80.7% in staff (IQR: 72.0–87.5) between May and June 2021.

**Table 1:** Baseline characteristics of included long-term care facilities with and without COVID-19 outbreaks across four time periods, England, November 2020–June 2021 (n = 330)^a^

Characteristics	Time period 1 Nov–Dec 2020			Time period 2 Jan–Feb 2021			Time period 3 Mar–Apr 2021				Time period 4 May–Jun 2021			
**Number of LTCF**	**n = 270**			**n = 210**			**n = 203**				**n = 322**			
**Number per LTCF^b^**	**Median**	**IQR**		**Median**	**IQR**		**Median**		**IQR**		**Median**		**IQR**	
Staff	43	2–58		47	3–60		48		35–63		45		34–62 (n = 319)	
Residents	38	28–50 (n = 269)		38	29–51 (n = 209)		36		28–49 (n = 201)		32		23–45 (n = 320)	
**Proportion per LTCF^c^**	**Median**	**IQR**		**Median**	**IQR**		**Median**		**IQR**		**Median**		**IQR**	
Female	68.5	61.7–74.1 (n = 269)		69.0	61.7–74.3 (n = 209)		68.9		61.5–76.3 (n = 201)		69.2		62.3–76.7 (n = 320)	
Male	31.5	25.9–38.3 (n = 269)		31.0	25.7–38.3 (n = 209)		31.1		23.7–38.5 (n = 201)		30.8		23.3–37.7 (n = 320)	
Aged > 80 years	75	65.6–82.5 (n = 269)		74.2	64.3–81.0 (n = 209)		73.5		63.9–80.8 (n = 201)		71.4		63.6–80 (n = 320)	
Beds for residents with dementia	54.7	49.6–76.9 (n = 50)		61.5	50–90.9 (n = 38)		59.6		48.8–100 (n = 38)		63.7		50–87.2 (n = 70)	
Staff vaccinated (first dose)	0	0–0		57.4	39.9–75.6 (n = 208)		76.3		67.6–84.5 (n = 201)		80.7		72.0–87.5 (n = 318)	
Residents vaccinated (first dose)	0	0–0		85.7	67.7–93.1 (n = 208)		88.0		78.0–93.5 (n = 201)		90.3		79.3–95.7 (n = 317)	
Staff vaccinated (second dose)	0	0–0		0	0–0		0		0–0		64.1		49.0–75.7 (n = 318)	
Residents vaccinated (second dose)	0	0–0		0	0–0		0		0–0		85.0		71.9–92.0 (n = 318)	
**Monthly turnover per LTCF^b^**	**Mean**	**IQR**		**Mean**	**IQR**		**Mean**		**IQR**		**Mean**		**IQR**	
Staff	3.3	1.7–4.9 (n = 81)		3.2	1.6–5.0 (n = 60)		2.9		1.7–4.5 (n = 63)		2.0		0–4.5 (n = 94)	
Resident	6.5	3.6–10.1 (n = 81)		5.6	3.5–7.7 (n = 60)		6.0		3.6–8.3 (n = 63)		7.9		4.3–13.2 (n = 94)	
**LTCF size (number of staff plus number of residents)**	**Total n = 269**			**Total n = 209**			**Total n = 201**				**Total n = 317**			
	**n**	**%**		**n**	**%**		**n**	**%**			**n**	**%**		
Small ( < 50)	42	15.6		26	12.4		26	12.9			48	15.1		
Medium (50–100)	148	55.0		111	53.1		105	52.2			175	55.2		
Large ( > 100)	79	29.4		72	34.5		70	34.8			94	29.7		
**LTCF type**	**Total n = 270**			**Total n = 210**			**Total n = 203**				**Total n = 322**			
	**n**		**%**	**n**		**%**	**n**			**%**	**n**			**%**
For-profit	180		66.7	157		74.8	136			67.0	225			69.9
Not-for-profit	59		21.9	27		12.9	43			21.2	60			18.6
Independent	31		11.5	26		12.4	24			11.8	37			11.5
**CQC rating**	**Total n = 269**			**Total n = 208**			**Total n = 201**				**Total n = 320**			
	**n**		**%**	**n**		**%**	**n**			**%**	**n**			**%**
Inadequate	6		2.2	5		2.4	2			1.0	6			1.9
Requires improvement	62		23.1	39		18.8	45			22.4	71			22.2
Good	191		71.0	157		75.5	148			73.6	233			72.8
Outstanding	10		3.7	7		3.4	6			3.0	10			3.1
**Region**	**Total n = 268**			**Total n = 208**			**Total n = 202**				**Total n = 320**			
	**n**		**%**	**n**		**%**	**n**			**%**	**n**			**%**
East Midlands	45		16.8	29		13.9	29			14.4	52			16.3
East of England	17		6.3	12		5.8	10			5.0	18			5.6
London	16		6.0	10		4.8	8			4.0	17			5.3
North East	15		5.6	13		6.3	15			7.4	21			6.6
North West	48		17.9	60		28.9	42			20.8	73			22.8
South East	42		15.7	21		10.1	36			17.8	40			12.5
South West	54		20.2	37		17.8	42			20.8	59			18.4
West Midlands	22		8.2	13		6.3	10			5.0	25			7.8
Yorkshire and the Humber	9		3.4	13		6.3	10			5.0	15			4.7
**Number of SARS-CoV-2 tests performed per LTCF^d^**	**Mean**		**SD**	**Mean**		**SD**	**Mean**			**SD**	**Mean**			**SD**
PCR, staff and residents	299.3		11.1	252.8		10.3 (n = 209)	259.3			10.6 (n = 202)	261.1			9.5
PCR, staff only	225.1		9.5 (n = 265)	181.7		8.4 (n = 204)	205.8			9.4 (n = 195)	207.5			8.1 (n = 312)
PCR, residents only	79.0		3.1 (n = 268)	75.4		3.8 (n = 209)	61.6			2.5 (n = 199)	61.8			2.3 (n = 313)
LFD, staff and residents	10.9		1.9 (n = 71)	181.3		11.1 (n = 202)	247.5			12.9 (n = 200)	219.4			9.8 (n = 309)
LFD, staff only	9.9		1.8 (n = 66)	181.2		11.1 (n = 199)	244.9			12.7 (n = 199)	214.9			9.5 (n = 309)
**SARS-CoV-2 tests performed which were positive per LTCF^e^**	**Mean**		**SD**	**Mean**		**SD**	**Mean**			**SD**	**Mean**			**SD**
PCR	2.32		0.29	3.29		0.50 (n = 209)	0.27			0.07 (n = 202)	0.38			0.31
LFD	0.66		0.48 (n = 71)	1.73		0.85 (n = 202)	0.05			0.01 (n = 200)	0.11			0.02 (n = 309)

CQC: care quality commission; IQR: interquartile range; LFD: lateral flow device; LTCF: long-term care facilities; PCR: polymerase chain reaction; SARS-CoV-2: severe acute respiratory syndrome coronavirus 2; SD: standard deviation.

^a^ Baseline characteristics of the LTCF assessed mid-way through each time period.

^b^ Median per LTCF, across all LTCF included in the time period (see Supplement).

^c^ Median proportion per LTCF, across all LTCF included in the time period.

^d^ Mean number of tests performed per LTCF after removal of duplicates, across all LTCF included in the time period when this type of testing was being performed in the LTCF (for example, LTCF with no LFD tests recorded were removed from the denominator).

^e^ Mean proportion of SARS-CoV-2-positive tests per LTCF after removal of duplicates, across all LTCF included in the time period when this type of testing was being performed in the LTCF (for example, LTCF with no LFD tests were removed from the denominator).

Note: The number of LTCF varied for some variables where LTCF information was not available for example, capacity tracker information pertaining to number of residents/staff/dementia beds was not consistently reported and monthly turnover data was not available for all LTCF. Number of LTCFs considered for these variables is supplied in the table in brackets as n.

The mean number of PCR tests performed per LTCF remained relatively stable (252.8–299.3), whereas mean LFD numbers increased substantially from 10.9 (standard deviation (SD): 1.9) LFD tests per LTCF between November and December 2020 to a peak of 247.5 (SD: 12.9) between March and April 2021, reflecting LFD changes in testing policy in England. Test positivity was highest between January and February 2021 (Table 1), consistent with national SARS-CoV-2 incidence [6].

## COVID-19 outbreaks

Between 1 November 2020 and 30 June 2021, there were 297 COVID-19 outbreaks across 330 LTCF, and 240 LTCF (73%) experienced at least one outbreak (Table 2). The mean number of outbreaks per LTCF was 0.90 (SD: 0.037). Between November 2020 and February 2021, when first dose vaccination coverage was lowest (57.4% and 85.7% of staff and residents, respectively, vaccinated mid-way through January and February 2021), over 50% of LTCF experienced a COVID-19 outbreak. By comparison, the proportion of LTCF experiencing outbreaks declined to 23/203 between March and April 2021, when over 75% of staff and over 85% of residents per LTCF had received first dose vaccination. The proportion further declined to 15/322 between May and June 2021, when 64.1% (IQR: 49.0–75.7) and 85.0% (IQR: 71.9–92.0) of staff and residents, respectively, had been fully vaccinated with two doses (Table 1 and Table 2).

**Table 2:** Characteristics of COVID-19 outbreaks in long-term care facilities across four time periods, England, November 2020–June 2021

Characteristics	Time period 1 Nov–Dec 2020		Time period 2 Jan–Feb 2021		Time period 3 Mar–Apr 2021		Time period 4 May–Jun 2021	
Number of outbreaks	145		114		23		15	
Number of LTCF with outbreaks	139		111		23		15	
Number of large outbreaks per time period^a^	31		9		0		0	
**Proportions**	**%**	**95% CI**	**%**	**95% CI**	**%**	**95% CI**	**%**	**95% CI**
Proportion of LTCF with outbreaks	51.5	45.5–57.4	52.9	46.1–59.6	11.3	7.6–16.5	4.7	2.8–7.6
Proportion of LTCF with large outbreaks^a^	11.5	8.2–15.9	4.3	2.2–8.1	0		0	
Proportion of outbreaks that only affected residents	9.0	5.3–14.9	7.0	3.5–13.5	17.4	6.3–39.7	13.3	2.9–44.0
Proportion of outbreaks that only affected staff	13.8	9.0–20.5	19.3	13.0–27.7	39.1	21.0–60.9	53.3	27.4–77.6
Proportion of outbreaks that affected residents and staff	77.2	69.6–83.4	73.7	64.8–81.0	43.5	24.3–64.8	33.3	13.4–61.8
**Characteristics**	**Mean (SD)**	**p value**	**Mean (SD)**	**p value**	**Mean (SD)**	**p value**	**Mean (SD)**	**p value**
Number of outbreaks per LTCF	0.54 (0.03)	Reference	0.54 (0.04)	0.8403	0.11 (0.02)	< 0.00001	0.05 (0.01)	< 0.00001
Number of large outbreaks per LTCF^a^	0.11 (0.02)	Reference	0.04 (0.01)	0.0047	0	Na	0	Na
Duration (days) of outbreak per LTCF^b^	63.0 (2.15)	Reference	48.04 (1.46)	< 0.00001	35.43 (1.47)	< 0.00001	34.07 (1.43)	< 0.00001
Proportion of residents who died of COVID-19 during an outbreak per LTCF^b^	5.09 (0.68) (n = 144)	Reference	3.32 (0.67)	0.0051	0.25 (0.17)	< 0.00001	0.16 (0.16)	0.0001
Proportion infected during an outbreak^b^	17.6 (1.51) (n = 144)	Reference	11.0 (1.10)	0.0046	3.58 (0.74)	< 0.00001	3.01 (0.68) (n = 14)	< 0.00001
Proportion of staff infected during outbreak^b^	15.6 (1.46)	Reference	9.05 (1.14)	0.0012	2.64 (0.43)	< 0.00001	3.61 (0.54) (n = 14)	0.0008
Proportion of residents infected during outbreak^b^	20.00 (1.87) (n = 144)	Reference	12.67 (1.47)	0.0229	4.10 (1.31)	< 0.00001	6.31 (4.21)	0.0002
Number of individuals infected per LTCF outbreak^b^	16.14 (1.39)	Reference	8.96 (0.77)	0.0039	3.30 (0.73)	< 0.00001	3.13 (0.46)	< 0.00001
Number of staff infected per LTCF outbreak^b^	7.38 (0.67)	Reference	4.04 (0.37)	0.0065	1.43 (0.19)	< 0.00001	1.80 (0.24)	0.0009
Number of residents infected per LTCF outbreak^b^	8.76 (0.80)	Reference	4.92 (0.52)	0.0054	1.87 (0.34)	< 0.00001	1.33 (0.54)	0.0001

CI: confidence interval; COVID-19: coronavirus disease; LTCF: long-term care facilities; Na: not applicable; SD: standard deviation.

^a^ Large outbreak defined as over one third of the LTCF being infected (staff and residents combined).

^b^ Total number of outbreaks analysed varied where total number of residents/staff in a LTCF was not available.

We used the Mann–Whitney test to calculate the p values and to compare means from time interval specified to mean values between November and December 2020.

**Figure 1:**
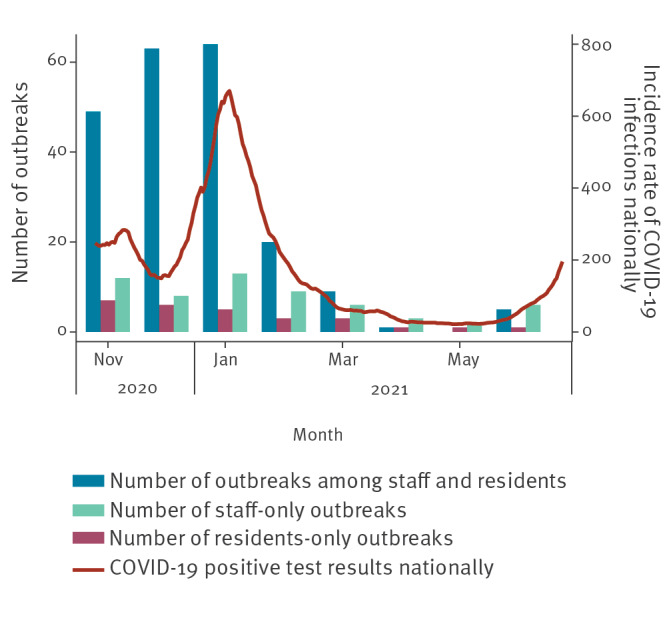
Number of COVID-19 outbreaks per month in long-term care facilities in residents only, staff only or staff and residents, England, November 2020–June 2021

**Figure 2:**
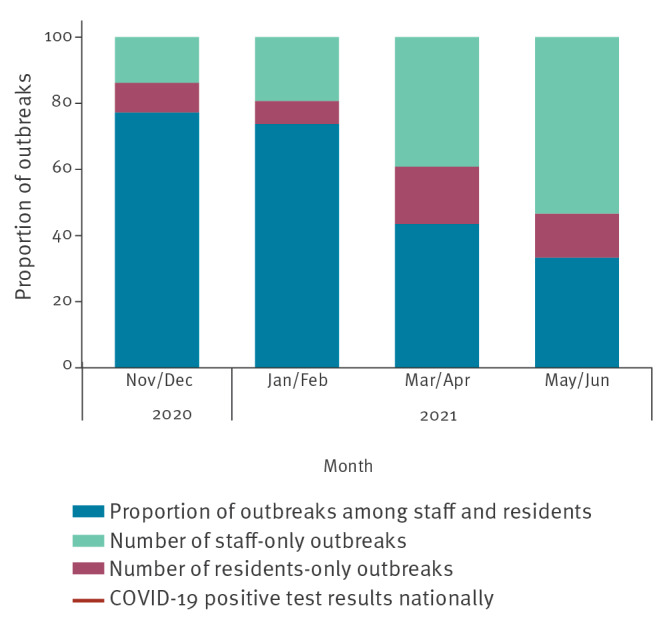
Proportion of residents-only, staff-only and staff and residents outbreaks in long-term care facilities, England, November 2020–June 2021

COVID-19: coronavirus disease; LTCF: long-term care facilities.

During November and December 2020, 112/145 outbreaks in LTCF were mixed, i.e. among staff and residents, dropping to 84/114 and 10/23 in subsequent time-periods and down to 5/15 by May and June 2021 (Figure 1 and Figure 2). Conversely, the proportion of outbreaks affecting only staff increased over time from 20/145 between November and December 2020 before vaccination roll-out, to 9/23 between March and April 2021, reaching 8/15 between May and June 2021. The proportion of outbreaks affecting only residents remained low compared to staff-only outbreaks, dropping to 8/114 between January and February 2021 and peaking at 4/23 between March and April 2021.

COVID-19: coronavirus disease.

Red line shows the national community incidence of SARS-CoV-2 [6].

**Figure 3:**
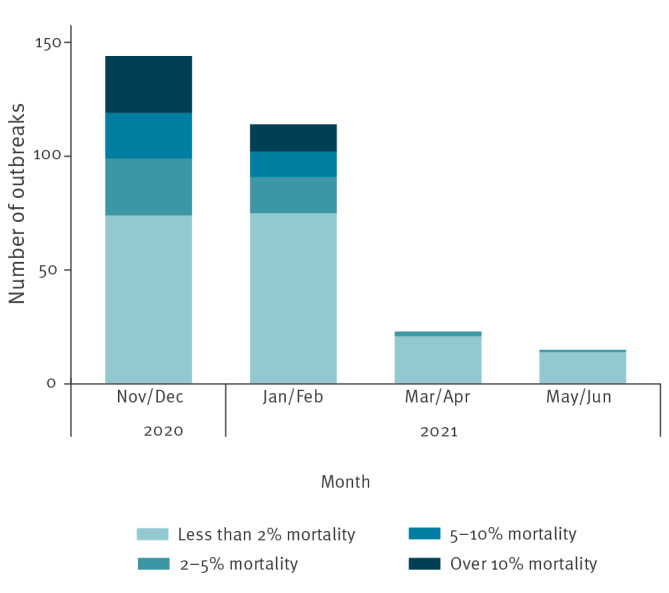
Proportion of residents who died in LTCF and corresponding number of outbreaks during the same time period over successive waves of the COVID-19 pandemic, England, November 2020–June 2021

COVID-19: coronavirus disease; LTCF: long-term care facilities.

Outbreak severity decreased as LTCF vaccination coverage increased, with an 80.6% reduction in the number of infected cases per outbreak and a 45.9% reduction in outbreak duration when comparing outbreaks between November and December 2020 with outbreaks between May and June 2021 (Table 2). The mean number of individuals infected per outbreak was slightly higher in residents than staff throughout, except between May and June 2021. The mean proportion of the LTCF infected during an outbreak decreased from 17.6% (SD: 1.51) between November and December 2020 to 3.01% (SD: 0.68) between May and June 2021, and was consistently higher in residents. The proportion of residents who died of COVID-19 or were infected with SARS-CoV-2 during an outbreak decreased over the study period; less than 5% of residents died of COVID-19 in LTCF experiencing outbreaks after March 2021 (Figure 3) and the proportion of individuals infected per outbreak decreased when first dose vaccination coverage exceeded 25% (see Supplementary Figure S1 and Table S3). There were no large outbreaks from March 2021 to the end of the study period (June 2021).

There was no substantial difference in results when outbreaks were considered to be over after 35 days without new positive tests vs 28 days (see Supplementary Table S2).

### Ethical statement

Ethical approval was obtained from the South Central Hampshire B Research Ethics Committee (reference number: 20/SC/0238).

## Discussion

From November 2020 to June 2021, as total LTCF vaccination coverage increased, the number of LTCF outbreaks and their severity decreased, with a concomitant increase in the proportion of staff-only outbreaks, while the attack rate per outbreak remained consistently higher for residents.

Our results suggest vaccination has had a major impact on the risk of LTCF outbreaks and supports evidence demonstrating vaccine effectiveness in LTCF residents and staff [7,8]. The change in outbreak characteristics may also be linked with broader policy changes (e.g. national lockdowns) and changing national incidence rates. Our study showed no clear effect following the emergence of more transmissible SARS-CoV-2 variants such as the Delta variant (Phylogenetic Assignment of Named Global Outbreak (Pango) lineage designation B.1.617.2), however, the last month of the study (June 2021) saw a slight increase in outbreak numbers relative to previous months, likely driven by increasing community incidence nationally [6].

The study identified a shift towards staff-only outbreaks, possibly associated with lower vaccination coverage in staff vs residents, greater infection risk associated with staff mixing outside of the LTCF, testing policy leading to increased testing of staff compared with residents, or possibly improved protection offered to residents by infection control measures. We also saw the proportion of resident-only outbreaks rising between January and February 2021 and between March and April 2021, although this remained far below the proportion of staff-only outbreaks over the same periods and the subsequent trend did not show an increase in resident-only outbreaks, so the importance appears minimal. Future studies with longer follow-up may explore this further. The higher attack rate in residents compared with staff is augmented by the lower total number of residents per LTCF compared with staff. However, given that more outbreaks affected only staff, similar average absolute numbers of infected staff and residents per outbreak indicate a relatively high number of resident cases in outbreaks that included residents relative to staff cases in outbreaks including staff. Further analysis is required to investigate the association between the larger absolute numbers for residents and LTCF size.

We saw trends in LTCF test positivity that mirrored national incidence rates and, while SARS-CoV-2 continues to circulate, this along with the shift towards staff only outbreaks supports the ongoing need for regular PCR/LFD testing of staff, although this may become less relevant following the introduction of mandatory vaccination of staff [9]. In addition, our findings support a review of resident testing because continuing to test residents regularly is pertinent considering the higher attack rate among residents, despite the shift towards staff-only outbreaks. Indeed, an increase in routine testing of residents may be warranted when community incidence is high to ensure cases are not missed.

Strengths of this study include the large, geographically representative LTCF cohort and access to regular routine PCR/LFD results, increasing the likelihood that outbreaks were correctly identified. Limitations include that, although we reported mortality data, we were unable to report on clinical severity as these data were unavailable. Another limitation is that defining outbreak end dates using testing data is challenging as residents are only tested monthly. Therefore, re-testing more than 28 days after outbreak commencement may be classified as a new outbreak, however, sensitivity analysis showed no substantial difference after extending the outbreak end-date definition. Finally, missing data resulted in numerous LTCFs being excluded during analysis, with the proportion of LTCFs excluded per time-period ranging from 0% to 82% across all variables. Further studies should model risk factors influencing the risk and severity of LTCF outbreaks to inform preventative measures to limit LTCF outbreaks.

## Conclusion

This study supports the ongoing national COVID-19 vaccination campaign in LTCF including vaccination of staff. It also supports continuation of testing in this setting while SARS-CoV-2 continues to circulate.

## Supplementary Data

191000 Supplement

## References

[1] McMichael TM Currie DW Clark S Pogosjans S Kay M Schwartz NG Epidemiology of Covid-19 in a long-term care facility in King County, Washington. N Engl J Med. 2020;382(21):2005-11. 10.1056/NEJMoa2005412 32220208 PMC7121761

[2] Krutikov M Palmer T Donaldson A Lorencatto F Forbes G Copas A Study protocol: understanding SARS-Cov-2 infection, immunity and its duration in care home residents and staff in England (VIVALDI). Wellcome Open Res. 2021;5:232. 10.12688/wellcomeopenres.16193.2 33564722 PMC7851710

[3] United Kingdom Government (GOV.UK). Department of Health and Social Care. Transparency data: Coronavirus (COVID-19) testing in care homes: statistics to 8 July 2020. London: GOV.UK; 16 Jul 2020. Available from: https://www.gov.uk/government/publications/coronavirus-covid-19-testing-in-care-homes-statistics-to-8-july-2020/coronavirus-covid-19-testing-in-care-homes-statistics-to-8-july-2020

[4] United Kingdom Government (GOV.UK). Department of Health and Social Care. Care home COVID-19 testing guidance: For testing of staff and residents. London: GOV.UK; 2021. Available from: https://assets.publishing.service.gov.uk/government/uploads/system/uploads/attachment_data/file/1021342/care-home-testing-guidance-england.pdf

[5] United Kingdom Health Security Agency (UKHSA). Guidance: COVID-19: epidemiological definitions of outbreaks and clusters in particular settings. London: UKHSA; 2020. Available from: https://www.gov.uk/government/publications/covid-19-epidemiological-definitions-of-outbreaks-and-clusters/covid-19-epidemiological-definitions-of-outbreaks-and-clusters-in-particular-settings

[6] United Kingdom Government (GOV.UK). Coronavirus (COVID-19) in the UK: download data. London: GOV.UK; 2021. [Accessed: 27 Jul 2021]. Available from: https://coronavirus.data.gov.uk/details/download

[7] Britton A Jacobs Slifka KM Edens C Nanduri SA Bart SM Shang N Effectiveness of the Pfizer-BioNTech COVID-19 vaccine among residents of two skilled nursing facilities experiencing COVID-19 outbreaks - Connecticut, December 2020-February 2021. MMWR Morb Mortal Wkly Rep. 2021;70(11):396-401. 10.15585/mmwr.mm7011e3 33735160 PMC7976620

[8] Shrotri M Krutikov M Palmer T Giddings R Azmi B Subbarao S Vaccine effectiveness of the first dose of ChAdOx1 nCoV-19 and BNT162b2 against SARS-CoV-2 infection in residents of long-term care facilities in England (VIVALDI): a prospective cohort study. Lancet Infect Dis. 2021;21(11):1529-38. 10.1016/S1473-3099(21)00289-9 34174193 PMC8221738

[9] United Kingdom Government (GOV.UK). Department of Health and Social Care. Coronavirus (COVID-19) vaccination of people working or deployed in care homes: operational guidance. London: GOV.UK; 2021. Available from: https://www.gov.uk/government/publications/vaccination-of-people-working-or-deployed-in-care-homes-operational-guidance/coronavirus-covid-19-vaccination-of-people-working-or-deployed-in-care-homes-operational-guidance

