# Supplementary material for: Changes in COVID-19 outbreak severity and duration in long-term care facilities following vaccine introduction, England, November 2020 to June 2021
Source: Euro Surveill. 2021 Nov 18;26(46):2100995. doi: 10.2807/1560-7917.ES.2021.26.46.2100995 (PMC8603404; doi:10.2807/1560-7917.ES.2021.26.46.2100995)
Supplement: Supplement [file 21-00995_GIDDINGS_Supplement.pdf]

# Supplementary materials: Changes in COVID-19 outbreak severity and duration in long-term care facilities following vaccine introduction, England, November 2020 to June 2021

*This supplementary material is hosted by Eurosurveillance as supporting information alongside the article "Changes in COVID-19 outbreak severity and duration in long-term care facilities following vaccine introduction, England, November 2020 to June 2021" on behalf of the authors who remain responsible for the accuracy and appropriateness of the content. The same standards for ethics, copyright, attributions and permissions as for the article apply. Supplements are not edited by Eurosurveillance and the journal is not responsible for the maintenance of any links or email addresses provided therein.*

**Supplementary information**

Supplementary Table S1: Data sources at individual, LTCF and national level.

Supplementary table S2: Characteristics of LTCF SARS-CoV-2 outbreaks per time period, where outbreak end definition was amended to no further positive tests reported for 35 days following the last positive

Supplementary table S3: LTCF Vaccination coverage by time period and outbreak statusSupplementary text S1: Data linkage methodology

Supplementary text S2: Staff/resident turnover analysis

Figure S1: Proportion infected during an outbreak by first dose vaccination coverage in staff and residents

**Supplementary Table S1: Data sources at individual, LTCF and national level.**

| Variable | Source | Utilisation |
|---|---|---|
| *Individual level* | | |
| Age, sex | National surveillance datasets | Care home characteristics |
| Vaccination status | National Immunisation Management Service (NIMS) | Care home characteristics[σ] |
| Hospital admissions and discharges | Hospital episode statistics (HES) | Care home characteristics[σ] |
| PCR/LFR results | Pillar 1 and Pillar 2 results | Identification of outbreak, measuring outbreak severity and Care home characteristics[σ] |
| *LTCF level* | | |
| CQC rating | Care Quality Commission (CQC) | Care home characteristics |
| Vaccination coverage | Capacity tracker | Care home characteristics* |
| Deaths | CQC mortality dataset[1] | Measure outbreak severity |
| LTCF characteristics (e.g. number dementia beds) | Capacity tracker | Care home characteristics |
| Turnover of staff and residents | Direct from care homes | Care home characteristics |
| *National level* | | |
| The national rate of new SARS-CoV-2 cases | Gov.uk[2] | Figures |

*Capacity tracker solely was utilized to determine vaccination coverage per LTCF as it enabled vaccination levels to vary; for example to decrease as new unvaccinated staff/residents entered the LTCF.

[σ]NIMS and HES data (alongside PCR/LFD tests) assisted in determining number of individuals present per LTCF per time-period

**Supplementary table S2:** Characteristics of LTCF SARS-CoV-2 outbreaks per time period, where an alternative outbreak end definition of no new positive tests for 35 days from last positive was applied

| | **Nov-Dec 2020** | | **Jan-Feb 2021** | | **Mar-April 2021** | | **May-June 2021** | |
|---|---|---|---|---|---|---|---|---|
| Number of LTCFs | 261 | | 191 | | 179 | | 314 | |
| Number of outbreaks per time period | 135 | | 102 | | 21 | | 15 | |
| Number of LTCFs with outbreaks per time period | 132 | | 100 | | 21 | | 15 | |
| Number of large outbreaks per time period* | 32 | | 9 | | 0 | | 0 | |
| | % (95%CI) | | % (95%CI) | | % (95%CI) | | % (95%CI) | |
| Proportion of care homes with outbreaks | 50.6 (44.5 – 56.6) | | 52.4 (45.2 – 59.4) | | 11.7 (7.8 – 17.4) | | 4.7 (2.9 – 7.8) | |
| Proportion of care homes with large outbreaks[a] | 12.3 (8.8 – 16.8) | | 4.7 (2.5 – 8.8) | | 0 | | 0 | |
| Proportion of outbreaks that only affected residents | 7.4 (4.0 – 13.3) | | 7.8 (3.9 – 15.0) | | 19.0 (6.9 – 42.9) | | 13.3 (2.9 – 44.0) | |
| Proportion of outbreaks that only affected staff | 11.9 (7.4 – 18.5) | | 17.6 (11.3 – 26.4) | | 38.1 (19.4 – 61.1) | | 53.3 (27.4 – 77.6) | |
| Proportion of outbreaks that affected residents and staff | 80.7 (73.1 – 86.6) | | 74.5 (65.1 – 82.1) | | 45.9 (23.0 – 65.3) | | 33.3 (13.4 – 61.8) | |
| | Mean (SD) | P value | Mean (SD) | P value | Mean (SD) | P value | Mean (SD) | P value |
| Number of outbreaks per care home | 0.52 (0.03) | comparator | 0.53 (0.46) | 0.7213 | 0.12 (0.02) | <0.00001 | 0.05 (0.01) | <0.00001 |
| Number of large outbreaks | 0.12 (0.02) | comparator | 0.05 (0.02) | 0.0058 | 0 | | 0 | |

| | | | | | | | | |
|---|---|---|---|---|---|---|---|---|
| per care home[a] | | | | | | | | |
| | | | | | | | | |
| Duration of outbreak per care home (days)[b] | 77.01 (2.59) | comparator | 59.31 (1.87) | <0.00001 | 41.71 (1.46) | <0.00001 | 39.53 (36.07) | <0.00001 |
| Proportion of residents dying from COVID-deaths during an outbreak per LTCF (%)[b] | 5.48 (0.73) (n=134) | comparator | 3.71 (0.74) | 0.011 | 0.27 (0.19) | <0.00001 | 0.16 (0.16) | 0.0001 |
| Proportion infected during an outbreak (%)[b] | 19.13 (1.58) (n=134) | comparator | 11.73 (1.18) | 0.0015 | 3.74 (0.80) | <0.00001 | 3.01 (0.68) (n=14) | <0.00001 |
| Proportion of staff infected during outbreak (%)[b] | 17.16 (1.54) | comparator | 9.50 (1.26) | 0.0002 | 2.66 (0.47) | <0.00001 | 3.61 (0.54) (n=14) | 0.0001 |
| Proportion of residents infected during outbreak (%)[b] | 21.72 (1.98) (n=134) | comparator | 13.54 (1.55) | 0.0191 | 4.40 (1.42) | <0.00001 | 6.31 (4.21) | 0.0001 |
| Number of individuals infected per LTCF outbreak[b] | 17.41 (1.45) | comparator | 9.29 (0.82) | 0.0005 | 3.38 (0.80) | <0.00001 | 3.13 (0.46) | <0.00001 |
| Number of staff infected per LTCF outbreak[b] | 8.01 (0.71) | comparator | 4.08 (0.40) | 0.0008 | 1.43 (0.20) | <0.00001 | 1.80 (0.24) | 0.0002 |
| Number of residents infected per LTCF outbreak[b] | 9.40 (0.84) | comparator | 5.22 (0.55) | 0.0033 | 1.95 (0.81) | <0.00001 | 1.33 (0.54) | <0.00001 |

CI: confidence interval; COVID-19: coronavirus disease; LTCF: long-term care facilities; Na: not applicable; SD: standard deviation.

[a] Large outbreak defined as over one third of the LTCF being infected (staff and residents combined)

p value calculated using Mann-Whitney's test to compare mean to mean value in Nov-Dec 2020

[b]Total number of outbreaks analysed varied where total number of residents/staff in a LTCF was not available.

**Supplementary table S3: LTCF Vaccination coverage by time-period and outbreak status**

| Vaccination coverage staff and residents | **Nov-Dec 2020** | | **Jan-Feb 2021** | | **Mar-April 2021** | | **May-June 2021** | | **Total** |
|---|---|---|---|---|---|---|---|---|---|
| | Outbreak | No outbreak | Outbreak | No outbreak | Outbreak | No outbreak | Outbreak | No outbreak | |
| <25% | 139 | 131 | 110 | 99 | 23 | 180 | 0 | 8 | 690 |
| 25-50% | 0 | 0 | 1 | 0 | 0 | 0 | 3 | 39 | 43 |
| 50-75% | 0 | 0 | 0 | 0 | 0 | 0 | 7 | 141 | 148 |
| 75-100% | 0 | 0 | 0 | 0 | 0 | 0 | 5 | 119 | 124 |

**Supplementary text S1: Data linkage methodology**

All datasets were analysed in the UCL Data Safe Haven using Stata v16.0.

**Individual level data linkage:** We retrieved results of PCR and LFD tests for staff and residents in participating LTCFs from 1 November 2020 to 30th June 2021. PCR and LFD results from the national testing programme (from routine and outbreak testing) are linked to the specific individual using a pseudo-identifier based on their unique National Health Service (NHS) number. These test results are then linked by CQC-IDs (Care Quality Commissions unique location ID) to specific homes; in this way LTCF residents and staff can be identified as being a member of a particular LTCF.

The NHS number-based pseudo-identifier was used to retrieve data from the National Immunisations Management System (NIMS) and hospital episode statistics (HES)

**LTCF level data linkage:** Capacity tracker was utilised to determine vaccination coverage per LTCF. Capacity tracker is a web-based tool which enables care homes across England to provide information on a daily basis pertaining to factors such as vaccination uptake (at aggregate level), vacancy information, PPE use and admission status. Data pertaining to the care homes included in the VIVALDI study was identified via CQC_IDs. For the purpose of this study, vaccination coverage was calculated from aggregate-level capacity tracker data as follows:

Staff vaccination coverage (dose 1) = vaccine_dose_1_staff_yes / total_staff

Resident vaccination coverage (dose 1) = vaccine_dose_1_residents_yes /total_occupied_beds

(Second dose coverage calculated similarly)

Staff and resident turnover data was sent in aggregate form directly to the VIVALDI study team from the larger LTCFs for analysis.

**Supplementary text S2: Staff/resident turnover analysis**

For each LTCF, the monthly resident and staff turnover rate was calculated using the following equation:

Monthly turnover per LTCF = Number leaving LTCF during month / ((total number at start of month + total number at end of the month)/2) X 100

For each time period in the study, the mean turnover per LTCF was then calculated. For example, for the November and December 2020 time period:

Mean turnover per LTCF during Nov/Dec 2020 = (November turnover per LTCF + December turnover per LTCF)/2

For the May/June time period, data were only available for the May time period.

For each time period in the study, the median turnover across *all* LTCF was then obtained:

Median turnover across all LTCF for time period X = median (mean turnover per LTCF during time period X for all LTCFs)

**Figure S1: Proportion of a LTCF (both staff and residents) infected during an outbreak by first dose vaccination coverage in staff and residents**

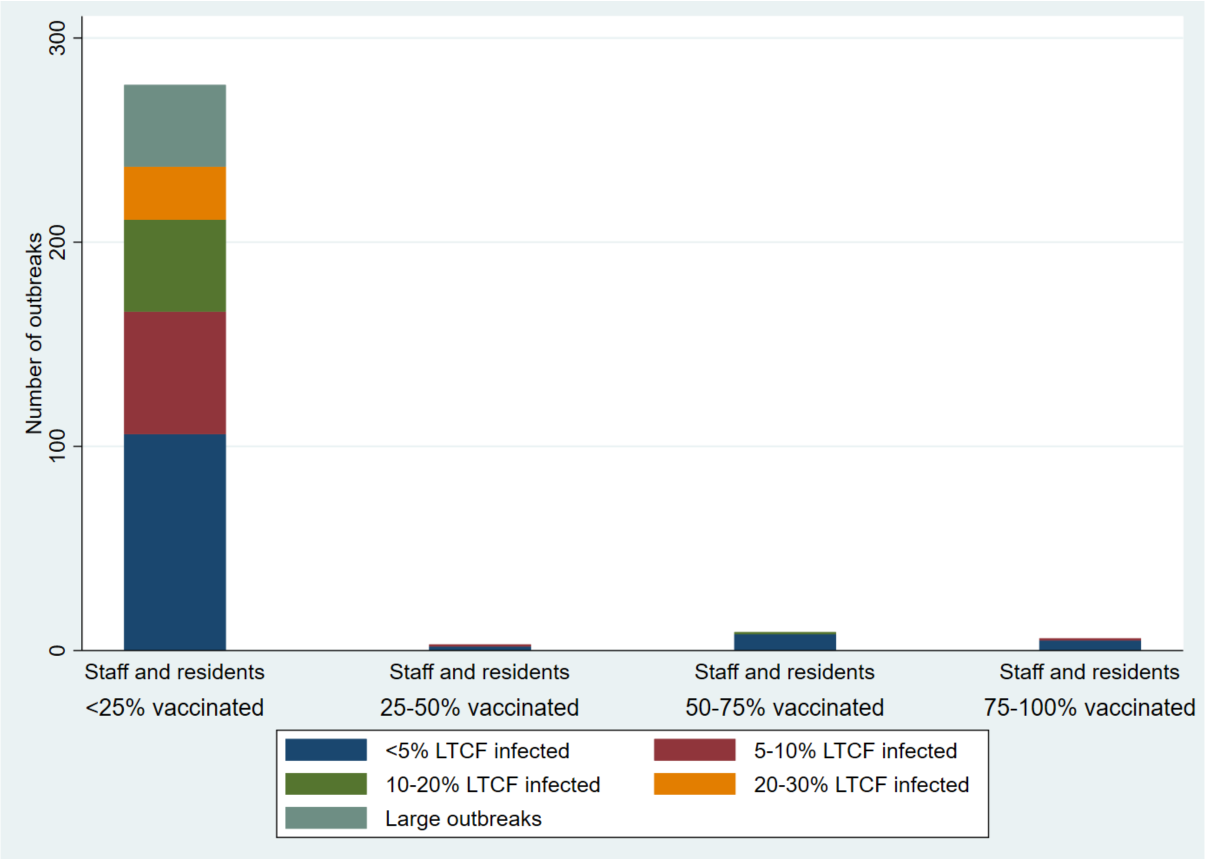


References

[1] Office for National Statistics, “Dataset: Number of deaths in care homes notified to the Care Quality Commission, England,” 2021. https://www.ons.gov.uk/peoplepopulationandcommunity/birthsdeathsandmarriages/deaths/datasets/numberofdeathsincarehomesnotifiedtothecarequalitycommissionengland (accessed Aug. 04, 2021).

[2] Gov.uk, “Coronavirus (COVID-19) in the UK: Download Data,” 2021. https://coronavirus.data.gov.uk/details/download (accessed Jul. 27, 2021).
